# Efficacy and safety of puerarin injection as an adjunctive therapy for chronic heart failure: a systematic review and meta-analysis

**DOI:** 10.3389/fphar.2025.1516059

**Published:** 2025-04-28

**Authors:** Hesong Fan, Jingrong Yang, Huaizhe Wang, Shuli Zong, Yiwei Qu, Kefei Wang, Fengyi Zhang, Xiao Li

**Affiliations:** ^1^ First Clinical Medical College, Shandong University of Traditional Chinese Medicine, Jinan, Shandong, China; ^2^ Affiliated Hospital of Shandong University of Traditional Chinese Medicine, Jinan, Shandong, China

**Keywords:** puerarin injection, chronic heart failure, meta-analysis, systematic review, traditional Chinese medicine

## Abstract

**Objective:**

Puerarin injection is used for the treatment of chronic heart failure (CHF). The objective of this study is to investigate the efficacy and safety of puerarin injection as an adjunct therapy for CHF through a meta-analysis and systematic review.

**Methods:**

We conducted a comprehensive literature search across eight databases, including PubMed, Embase, Web of Science, and Cochrane Library, up to 1 March 2025, to identify the required randomized controlled trials (RCTs). Subsequently, we assessed the included studies according to the principles outlined in the Cochrane Handbook and performed a meta-analysis using RevMan 5.4 and Stata 12.0.

**Results:**

A total of 29 RCTs were included, comprising 2,480 patients, with 1,251 in the Puerarin group and 1,229 in the control group. The meta-analysis demonstrated that puerarin injection combined with conventional medication significantly improved cardiac function parameters in chronic heart failure patients compared to pharmacotherapy alone, including left ventricular ejection fraction (MD = 6.22, 95% CI [3.11, 9.33], *P* < 0.01), cardiac output (MD = 0.45, 95% CI [0.35, 0.55], *P* < 0.01), and stroke volume (MD = 3.29, 95% CI [2.02, 4.57], *P* < 0.01), while reducing left ventricular end-diastolic diameter (MD = −0.83, 95% CI [−1.24, −0.42], *P* < 0.01). The combination therapy demonstrated both a significantly increased total effectiveness rate (RR = 1.26, 95% CI [1.21, 1.31], *P* < 0.01) and improved hemodynamic parameters, along with favorable modulation of oxidative stress markers evidenced by elevated superoxide dismutase, glutathione peroxidase, and catalase levels concomitant with reduced lipid peroxidation and malondialdehyde concentrations.

**Conclusion:**

This meta-analysis suggests that adjunctive puerarin injection with conventional therapy may provide comprehensive benefits for chronic heart failure management, including improved clinical outcomes, enhanced cardiac function, attenuated ventricular remodeling, optimized hemodynamic performance, and reduced oxidative stress, while maintaining a safety profile comparable to conventional therapy. However, due to the suboptimal quality and some degree of heterogeneity in the existing evidence, there is a need for more high-quality studies to provide more reliable evidence for its future clinical application.

**Systematic Review Registration:**

https://www.crd.york.ac.uk/PROSPERO/, identifier CRD42022371583.

## 1 Introduction

Chronic heart failure (CHF) is a complex and persistent clinical syndrome resulting from various factors leading to abnormalities in cardiac structure and function (McDonagh et al., 2021). Common symptoms of CHF include dyspnea, fluid retention, and weakness (Dick and Epelman, 2016).

Global Burden of Disease (GBD) data indicate that from 1990 to 2021, the global prevalence of heart failure (HF) and Years Lived with Disability (YLD) burden have significantly increased. Heart failure is more prevalent among the elderly population (Ran et al., 2025). According to data from the National Health and Nutrition Examination Survey (NHANES) conducted between 2017 and 2020 (Ss et al., 2024), approximately 6.7 million Americans aged 20 and older have heart failure. It is projected that from 2012 to 2030, the prevalence of heart failure will increase by 46%, affecting over 8 million individuals aged 18 and older. Heart failure represents a significant public health issue, imposing a considerable burden on individuals as well as healthcare systems.

The pathophysiological core of CHF is characterized by myocardial remodeling as the structural foundation and neurohormonal hyperactivation as the primary driver, initiating a multidimensional cascade of pathophysiological events. This progression encompasses oxidative stress-mediated mitochondrial dysfunction, dysregulated inflammatory cascades, calcium homeostasis disruption impairing excitation-contraction coupling, and maladaptive metabolic reprogramming, ultimately culminating in a self-perpetuating vicious cycle (McDonagh et al., 2023).

CHF management presents significant therapeutic challenges due to its pathophysiological complexity, necessitating lifelong pharmacological intervention. Current guideline-recommended pharmacotherapy encompasses multiple drug classes: diuretics for volume management, renin-angiotensin system inhibitors (including ACE inhibitors/ARBs/ARNIs), beta-blockers, mineralocorticoid receptor antagonists (MRAs), sodium-glucose cotransporter 2 inhibitors (SGLT2i), soluble guanylate cyclase stimulators, ivabradine for heart rate control, and digitalis glycosides (Ostrominski et al., 2024; Chinese Society of Cardiology et al., 2024).

However, despite many CHF patients receiving maximal treatment from existing therapeutic regimens, their prognosis remains poor (Xanthakis et al., 2016). Patients with heart failure often require long-term medication and frequent hospitalizations, which has become a significant social and healthcare issue (Lesyuk et al., 2018). In addition, the use of these medications is limited by their side effects, particularly in patients with comorbid systemic diseases (Mullens et al., 2022), such as electrolyte imbalances, renal impairment, bradycardia, hypotension, and arrhythmias (Williams and Oakeshott, 2014). Thus, it is essential to explore a safe and effective treatment option to complement existing CHF therapies.

Pueraria lobata (Willd.) Ohwi was recorded in the earliest Chinese herbal monograph “Shennong Ben Cao Jing” and included in the Pharmacopoeia of the People’s Republic of China (2020 Edition) (Pharmacopoeia Commission of the People’s Republic of China, 2020b). In traditional Chinese medicine (TCM), Pueraria lobata (Willd.) Ohwi is recognized for its dual pharmacological properties: body fluid generation promotion (Shengjin), as well as dredging meridians and activating collaterals (Tongjing Huoluo). We also verified this information through the KEWSCINCE database (http://mpns.kew.org). Pueraria lobata (Willd.) Ohwi is primarily indicated for the prevention and treatment of cardiovascular diseases, as documented in clinical studies (Shi et al., 2023; Wan et al., 2024).

Puerarin (7,4′-dihydroxyisoflavone-8-pyran glycoside) is an isoflavone derivative and one of the main bioactive components of the TCM Pueraria. It is extracted from Pueraria lobata. We checked the chemical structure of Puerarin on the Pharmacopoeia of the People’s Republic of China (2020 edition) (Pharmacopoeia Commission of the People’s Republic of China, 2020a) and compared and confirmed it on the website of NATIONAL LIBRARY OF MEDICINE. Due to its poor water and fat solubility, puerarin has limited oral absorption and low bioavailability (approximately 7% for oral administration), which often necessitates intravenous administration in clinical settings (Zhang, 2019).

Modern pharmacological studies have further uncovered the therapeutic potential of puerarin in heart failure (Wang et al., 2022). A recent study by Pan et al. (2024) demonstrated that puerarin acts as an effective anti-heart failure agent by inhibiting p38 mitogen-activated protein kinase and its downstream effector NHE1 (sodium-hydrogen exchanger 1). This dual inhibition mitigates mitochondrial damage and reduces the expression of TGF-β1 and pro-inflammatory cytokines, thereby suppressing cardiac fibrosis and ultimately improving heart failure outcomes. Puerarin stimulates protective autophagy via the 14-3-3γ/PKCε pathway, thereby mitigating myocardial damage caused by lipopolysaccharide and doxorubicin (Peng et al., 2022; Peng et al., 2022). Puerarin attenuates cardiomyocyte injury and improves cardiac function by selectively inhibiting acid sphingomyelinase (ASM)-mediated ceramide signaling (Li et al., 2025). Puerarin exerts cardioprotective effects by upregulating the KLF4/Mzb1 pathway, thereby alleviating oxidative stress and endoplasmic reticulum stress (Han et al., 2021). Furthermore, puerarin targets the PGAM5-VDAC1 axis to modulate mitophagy, inhibiting lipopolysaccharide (LPS)-induced necroptosis in cardiomyocytes and potentially reversing mitochondrial pathway-related cardiac damage (Zhuang et al., 2024). Puerarin injection was approved for marketing in China in the 1990s. It can improve cardiac hemodynamics and myocardial metabolism, as well as cerebral microcirculation, and is commonly used for cardiovascular and cerebrovascular diseases (Zhou et al., 2014).

Although numerous preclinical studies in recent years have demonstrated puerarin’s positive effects in cardiomyocyte protection, cardiac remodeling improvement, and cardiac function enhancement, and clinical trials of puerarin injection for CHF have also shown promising results, there remains a critical lack of high-quality randomized controlled trials (RCTs) to validate its efficacy in chronic heart failure patients. Conversely, some studies have raised concerns regarding potential adverse effects (Xie et al., 2018).

Systematic reviews possess a greater capacity to detect the impact of interventions on outcomes compared to individual studies, as the combined confidence interval (CI) can be more precise than that of most individual studies (Hernandez et al., 2020). Existing meta-analyses evaluating the efficacy and safety of puerarin in adjuvant treatment for chronic heart failure (Lian et al., 2016) have several limitations: The study only performed meta-analytical pooling without concurrent systematic review, leading to unidimensional evidence integration; Included randomized controlled trials (RCTs) were limited to the 1997–2013 timeframe, failing to account for advancements in the past decade; Insufficient sample sizes and absence of GRADE evidence quality assessment compromised robustness; Outcome measures were restricted, with inadequate discussion of adverse event profiles and substantial heterogeneity across included studies. Collectively, these issues undermine the clinical persuasiveness of current conclusions, leaving the evidence for puerarin injection’s therapeutic benefits in CHF management ambiguous. Therefore, our study aims to conduct a comprehensive meta-analysis and systematic review of puerarin injection’s efficacy and safety in adjuvant CHF treatment, providing updated evidence for clinical decision-making.

## 2 Methods

The systematic review and meta-analysis (SR/MA) will be conducted based on the guidelines outlined in the Cochrane Handbook (Hernandez et al., 2020) and the Preferred Reporting Items for Systematic Reviews and Meta-Analyses (PRISMA) statement (Supplementary Table S1) (Page et al., 2021), along with other high-quality research methodologies. This overview protocol has been registered on the PROSPERO website (Registration number: CRD42022371583).

### 2.1 Inclusion and exclusion criteria

#### 2.1.1 Inclusion criteria for literature

1 Study Type: Clinical randomized controlled trials, with no language restrictions.
2 Participants: Patients with clinically diagnosed CHF according to national or international standards, regardless of race, nationality, belief, region, age, or gender, classified as NYHA functional classes I–IV.
3 Search Timeframe: From the creation date of each database to 1 March 2025.
4 Intervention Measures: the control group will receive standard conventional treatment (McDonagh et al., 2021; Chinese Society of Cardiology et al., 2024), including cardiac inotropic therapy, diuretics, vasodilators, and ventricular remodeling modification, etc. In comparison to the control group, the experimental group will receive puerarin injection in addition to standard treatment.
5 Outcome Indicators: Primary outcome indicators include left ventricular ejection fraction (LVEF), Improvement in NYHA functional classification (the total effective rate) and adverse reactions. Secondary outcome indicators include Cardiac Output (CO), left ventricular end-diastolic diameter (LVEDD), left ventricular stroke volume (SV), cardiac index (CI), blood rheology examinations (low shear viscosity, high shear viscosity, plasma viscosity, platelet aggregation rate, fibrinogen), and oxidative stress status (superoxide dismutase, glutathione peroxidase, catalase, plasma lipid peroxides, malondialdehyde). The total effective rate was operationally defined as the proportion of patients achieving therapeutic response, which required concurrent fulfillment of both criteria: (1) resolution of CHF-related clinical symptoms (dyspnea, fatigue) and physical signs (peripheral edema, pulmonary rales), and (2) improvement in cardiac functional capacity by ≥1 NYHA class. Non-responders were characterized by either persistence/worsening of clinical manifestations or failure to achieve ≥1 NYHA class improvement despite therapeutic intervention.

#### 2.1.2 Exclusion criteria for literature

1 Studies involving patients with acute heart failure.
2 Animal experiments, mechanistic studies, case reports, expert reviews, meta-analyses, and systematic reviews, etc.
3 Studies with incomplete data that cannot provide valid endpoint information or those containing significant errors that remain unresolved even after contacting the original authors.
4 Publications with potential duplicate submissions.
5 Studies affected by confounding factors that cannot be controlled for, such as the combination of interventions with other TCM treatments.

### 2.2 Search strategy

Two researchers (JY and HW) conducted independent literature searches, screening eligible RCTs based on pre-defined inclusion and exclusion criteria. Discrepancies arising during the study selection process were resolved through discussion with a third party (XL). Searches were performed in eight databases: PubMed, Embase, Web of Science, Cochrane Library, CNKI, Chongqing VIP, WanFang Database, and China Biological Medicine Database (SinoMed), covering the period from each database’s inception to 1 March 2025. The search strategy included the use of MeSH terms and free text, with the following keywords: “cardiac failure,” “heart failure,” “CHF,” “chronic heart failure,” “puerarin,” “puerarin injection,” “kakonein.” Additionally, we manually reviewed relevant references to supplement the search results. PubMed search query as follows (“Puerarin” [Mesh] OR Puerarin [tiab] OR Kakonein [tiab] OR “Puerarin injection” [tiab]) AND (“Heart Failure” [Mesh] OR “Cardiac Failure” [Mesh] OR (“heart failure” [tiab] OR “cardiac failure” [tiab] OR CHF [tiab] OR “chronic heart failure” [tiab])). Supplementary Table S1 presents the overall search strategy.

### 2.3 Qualification assessment and data extraction

Based on our established inclusion and exclusion criteria, two researchers (JY and HW) independently screened the literature using EndNote software. After removing duplicate entries, the researchers performed an initial screening by reading the titles and abstracts of selected literature to identify potentially valuable studies. Subsequently, the full texts of these studies were obtained, and they were carefully read to further determine compliance with the inclusion criteria. In cases of disagreement between the two researchers during the literature screening process, resolution was achieved through consultation with a third party (KW). Next, the two researchers (JY and HW) independently extracted data using standardized data extraction forms. The following specific characteristics were extracted from each identified study: basic information about the study (author names, title, publication date); detailed information about the included patients (sample size, gender, mean age, treatment duration, intervention measures); quality information of the RCT (Jadad score); outcome indicators and main conclusions. This process ensured the accuracy and consistency of data extraction.

### 2.4 Risk of bias assessment

All included studies will be assessed by two researchers (SZ and YQ) according to the guidelines in the Cochrane Handbook. We will evaluate the quality of the studies based on the following seven domains: 1. Random sequence generation; 2. Allocation concealment; 3. Blinding of participants and personnel; 4. Blinding of outcome assessors; 5. Completeness of outcome data; 6. Selective reporting of outcomes; 7. Other sources of bias. The quality of each randomized controlled trial will be classified as “high risk,” “low risk,” or “unclear risk.” In cases of disagreement between the two researchers during the assessment process, discrepancies will be resolved through discussion with a third party (KW) to reach a final conclusion.

Additionally, two reviewers (SZ and YQ) evaluated the quality of included randomized controlled trials using the Jadad scale. The total score ranges from 0 to 7, with 4–7 indicating high-quality studies and 1–3 indicating low-quality studies. The criteria are defined as follows: 1. Random sequence generation Adequate: Computer-generated random numbers or similar methods (2 points). Unclear: Randomized trial without describing allocation method (1 point). Inadequate: Alternating allocation (e.g., odd/even numbers) (0 points); 2. Allocation concealment Adequate: Centralized/pharmacy-controlled allocation, sequentially numbered containers, on-site computer systems, sealed opaque envelopes, or other methods preventing prediction of assignments (2 points). Unclear: Mention of random number tables or allocation schemes without details (1 point). Inadequate: Alternating allocation, case numbers, day-of-week assignments, open random number lists, or unsealed envelopes (0 points). Not used: 0 points; 3. Blinding Adequate: Identical placebos or comparable blinding methods (2 points). Unclear: Statement of blinding without methodology (1 point). Inadequate: No blinding or flawed blinding (e.g., comparing tablets vs injections) (0 points); 4. Withdrawals and dropouts Described: Reported numbers and reasons for withdrawals/dropouts (1 point). Not described: No mention of withdrawals/dropouts (0 points).

### 2.5 Data statistics and analysis

Data analysis will be conducted using RevMan 5.4 software provided by the Cochrane Collaboration, supplemented by Stata 12.0 software. The heterogeneity among studies will be assessed using the Chi-squared test. When *P* ≥ 0.05 and *I* ^2^ ≤ 50%, it indicates low heterogeneity, and we will use a fixed-effect model for the meta-analysis; conversely, if *P* < 0.05 or *I* ^2^ > 50%, it indicates significant heterogeneity, and we will employ a random-effects model. To assess publication bias, we will use funnel plots for visual analysis. If results indicate high heterogeneity or further exploration is needed, subgroup and sensitivity analyses will be conducted to investigate the sources of heterogeneity and verify the robustness of the results. Finally, we will evaluate the quality of the meta-analysis using GRADEprofiler software.

## 3 Results

### 3.1 Literature search and screening results

**FIGURE 1:**
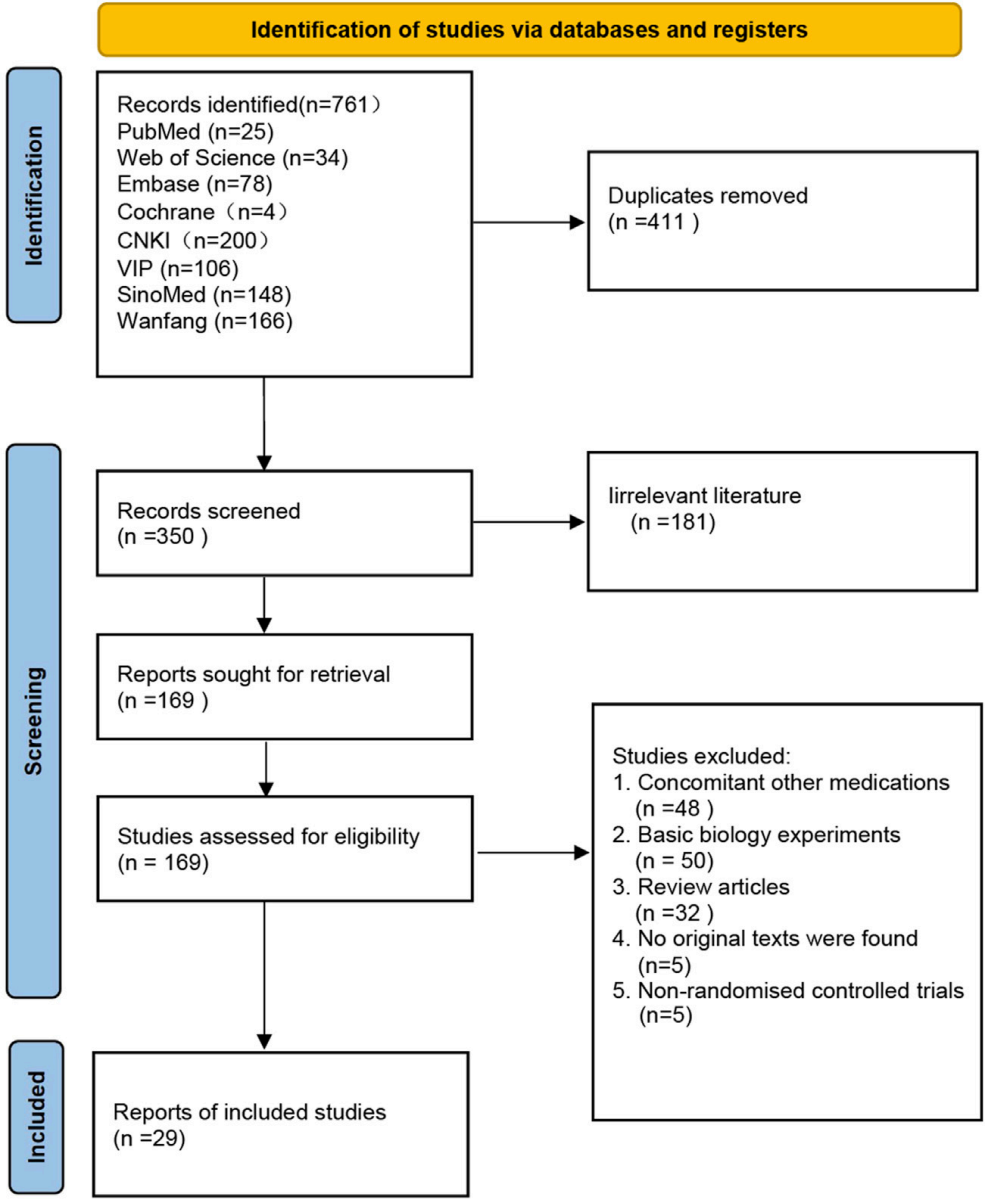
Flow diagram of studies selection process.

A total of 761 articles were retrieved from eight literature databases. After removing duplicate entries, 350 articles remained. We performed an initial screening by reading the titles and abstracts of these articles, Excluded 181 irrelevant articles, resulting in 169 articles selected for full-text screening. Following the evaluation based on the pre-defined inclusion and exclusion criteria, a total of 29 articles were ultimately confirmed for inclusion in the study. The screening flowchart can be found in Figure 1.

### 3.2 Study characteristics

All 29 RCTs (Xu, 1999; Duan et al., 2000; Cui and Xu, 2002; Zhang, 2003; Wang, 2003; Liu et al., 2004; Zhang and Lin, 2004; Liu et al., 2005; Liang et al., 2005; Jiao, 2005; Yu and Liu, 2006; Gong and Wang, 2006; Xu and Zhao, 2006; Su, 2006; Cai and Liu, 2006; Guo et al., 2006; Lu, 2006; Li, 2007; Yan et al., 2007; Lin et al., 2008; Tian, 2008; Shi and Lu, 2008; Sang, 2009; Lu, 2009; Hou, 2010; Pan, 2011; Wang and Xie, 2013; Zhao, 2016; Zeng et al., 2019) were published in Chinese journals, with publication dates ranging from 2003 to 2019. Their Jadad scores, ranging from 3 to 4, indicate that the studies have lower quality. The number of CHF patients in the included RCTs varied from 40 to 184, totaling 2,480 patients with 1,251 in the Puerarin group and 1,229 in the control group. The duration of treatment ranged from 10 to 30 days, with doses of puerarin injection in the treatment group varying from 50 mg to 600 mg per day. Detailed information is provided in Table 1.

**TABLE 1:** Characteristics of the included studies.

ID	Object of observation			Intervention		Treatment	Outcomes	Jadad score	NYHA (T/C)	
	Total sample (T/C)	Sex [T (M/F): C (M/F)]	Age (T: C)	T	C				T	C
Xu (1999)	(30/38)	(20/10): (28/10)	(50–78): (55–82)	CGT + PI (500 mg iv drip qd)	IT + DT + VT + OT et al.	14d	Clinical efficacy	3	III:18; IV:12	III:28; IV:10
Duan et al. (2000)	78 (38/40)	(19/19): (22/18)	(54.2 ± 8.2): (52.6 ± 9.45)	CGT + PI (400 mg iv drip qd)	IT + DT + VT et al.	10d	LVEF, Adverse events	4	II:21; III:46; IV:11	
Cui and Xu (2002)	(40/40)	(35/5): (36/4)	(62.3): (61.8)	CGT + PI (500 mg iv drip qd)	IT + DT + VT et al.	21d	LVEF, LVEDD	3	II:12; III:20; IV:8	II:11; III:22; IV:7
Wang (2003)	(48/48)	(28/20): (28/20)	(56): (56)	CGT + PI (400 mg iv drip qd)	IT + DT + VT	14d	Clinical efficacy, LVEF	3	II:25; III:56; IV:15	
Zhang (2003)	(60/60)	(78/42) (no details)	(65.0 ± 8.8) (no details)	CGT + PI (300 mg iv drip qd)	IT + DT + VT	15d	Clinical efficacy	3	III:79; IV:41	
Liu et al. (2004)	78 (39/39)	(57/21) (no details)	(74 ± 3) (no details)	CGT + PI (300 mg iv drip qd)	IT + DT + VT	14d	Clinical efficacy, SOD, GSH-Px, CAT, LPO, MDA	3	IV:78	
Zhang and Lin (2004)	(39/43)	(28/15): (26/13)	(66.9): (65.8) (no details)	CGT + PI (400 mg iv drip qd)	IT + DT + VT + OT et al.	14d	Clinical efficacy	3	II:9; III:22; IV:12	II:11; III:19; IV:9
Jiao (2005)	(50/52)	(35/15): (26/13)	(70.2 ± 10.3): (73.6 ± 11.1)	CGT + PI (400 mg iv drip qd)	IT + DT + VT + OT + NS et al.	10d	Clinical efficacy, Adverse events	4	II:3; III:50; IV:49	
Liang et al. (2005)	(42/42)	(17/11): (29/13)	(59.2 ± 12.1): (60.1 ± 11.2)	CGT + PI (500 mg iv drip qd)	RT	10d	Clinical efficacy, Hemorheology, Adverse events	3	III:22; IV:20	III:23; IV:19
Liu et al. (2005)	76 (38/38)	(56/20) (no details)	72 ± 4 (no details)	CGT + PI (300 mg iv drip qd)	IT + DT + VT	14d	Clinical efficacy, SOD, GSH-Px, CAT, LPO, MDA	3	IV:76	
Cai and Liu (2006)	(30/30)	(18/12): (20/10)	(61.21 ± 10.17): (59.86 ± 9.72)	CGT + PI (400 mg iv drip qd)	RT	14d	Clinical efficacy, CO, LVEF, SV, CI, Hemorheology, Adverse events	3	NR	
Gong and Wang (2006)	(43/43)	(24/19): (25/18)	(54 ± 8): (53 ± 9)	CGT + PI (400 mg iv drip qd)	IT + DT + VT + OT et al.	14d	Clinical efficacy, CO, LVEF, LVEDD, SV, CI, Adverse events	3	III:57; IV:29	
Guo et al. (2006)	82 (43/39)	(22/21): (20/19)	(53.6 ± 7.8): (54.2 ± 8.68)	CGT + PI (400 mg iv drip qd)	IT + DT + VT et al.	14d	Clinical efficacy, LVEF	3	II:26; III:42; IV:14	
Lu (2006)	(20/20)	(25/15) (no details)	65 (no details)	CGT + PI (50 mg iv drip qd)	IT + DT + VT	15d	Clinical efficacy, LVEF, CI	3	II:5; III:23; IV:12	
Su (2006)	(54/54)	NR	47.8 (no details)	CGT + PI (400 mg iv drip qd)	RT	10d	Clinical efficacy, LVEF, SV, CI, Adverse events	3	III-IV:108	
Xu and Zhao (2006)	(100/84)	(60/40): (52/32)	(63.8): (61.1)	CGT + PI (500 mg iv drip qd)	IT + DT + VT + OT et al.	10–20d	Clinical efficacy, Adverse events	3	II:42; III:39; IV:19	II:37; III:32; IV:15
Yu and Liu (2006)	(39/39)	(21/18): (20/19)	(63.5): (63)	CGT + PI (500 mg iv drip qd)	IT + DT + OT et al.	14d	Clinical efficacy, Adverse events	3	III:25; IV:14	III:26; IV:13
Li (2007)	(30/30)	NR	(60–80) (no details)	CGT + PI (500 mg iv drip qd)	IT + DT + VT + NS et al.	14d	Clinical efficacy, CO, LVEF, SV, CI, Adverse events	3	III-IV:60	
Yan et al. (2007)	(28/28)	(18/10): (19/9)	(56 ± 8): (55 ± 9)	CGT + PI (500 mg iv drip qd)	IT + DT + VT + OT et al.	14d	LVEF, LVEDD, Adverse events	3	II:16; III:12	II:18; III:10
Lin et al. (2008)	56 (28/28)	(17/11): (18/10)	(60.57 ± 9.86): (58.92 ± 9.65)	RT + PI (400 mg iv drip qd)	RT	14d	Clinical efficacy, Hemorheology, Adverse events	3	NR	
Shi and Lu (2008)	(43/43)	(51/35) (no details)	53.2 ± 8.23 (no details)	CGT + PI (600 mg iv drip qd)	IT + DT + VT et al.	14d	Clinical efficacy, CO, LVEF, LVEDD, SV, CI, Adverse events	3	III:57; IV:29	
Tian (2008)	(64/55)	(47/17): (42/13)	(60.9): (60.1)	CGT + PI (200–400 mg iv drip qd)	RT	15d	Clinical efficacy, Hemorheology	3	I:16; II:27; III:21	I:8; II:30; III:17
Lu (2009)	56 (28/28)	(17/11): (15/13)	(70.5 ± 5.0): (69.5 ± 9.0)	CGT + PI (400 mg iv drip qd)	IT + DT + VT + OT + NS et al.	14d	Clinical efficacy, Adverse events	3	II:3; III:38; IV:15	
Sang (2009)	(43/42)	(27/16): (23/19)	(20–72): (22–76)	CGT + PI (250 mL iv drip qd)	IT + DT + VT	14d	Clinical efficacy, LVEF, CO, SV, CI	3	II:13; III:24; IV:6	II:12; III:22; IV:8
Hou (2010)	(38/30)	(24/14): (21/9)	(35–70): (29–79)	CGT + PI (400 mg iv drip qd)	IT + DT + VT et al.	20d	Clinical efficacy	3	II:20; III:15; IV:3	II:13; III:11; IV:6
Pan (2011)	(28/28)	(19/9): (20/8)	(63.7 ± 2.1): (65.2 ± 1.9)	CGT + PI (400 mg iv drip qd)	IT + DT + OT + NS et al.	14d	Clinical efficacy, Hemorheology	3	NR	
Wang and Xie (2013)	(74/74)	(17/11): (15/13)	(39–74): (36–74)	CGT + PI (250 mL iv drip qd)	IT + DT + VT	28d	Clinical efficacy, Adverse events	3	II:26; III:26; IV:22	II:28; III:24; IV:22
Zhao (2016)	(49/49)	(30/19): (28/21)	(59.2 ± 11.7): (58.7 ± 12.6)	CGT + PI (500 mg iv drip qd)	IT + DT + VT et al.	20d	Clinical efficacy, LVEF, CO, Adverse events	3	II-IV:98	
Zeng et al. (2019)	(45/45)	(24/21): (25/20)	(67.04 ± 10.85): (67.28 ± 11.64)	CGT + PI (500 mg iv drip qd)	IT + DT + VT + Tanshinone IIA sulfonate sodium	30d	Clinical efficacy, LVEF, CO, LVEDD	4	NR	

Abbreviations: F, female; M, male; NR, not reported; T, Puerarin group; C, Control group; CGT, Control group treatment; RT, Routine Treatment; IT, Inotropic Therapy; DT, Diuretic Therapy; VT, Vasodilator Therapy; PI, Puerarin Injection; OT, Oxygen Therapy; NS, Nutritional Support.

**FIGURE 2:**
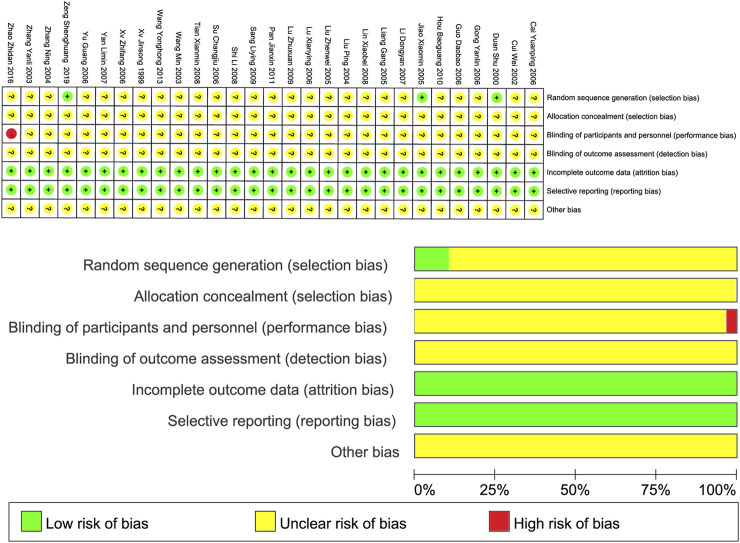
Risk of bias assessment for included studies.

Three studies (Duan et al., 2000; Jiao, 2005; Zeng et al., 2019) used random number tables for randomization, rated as low risk of bias. The remaining 26 studies only mentioned random grouping, so their risk of bias was unclear. One study (Zhao, 2016) explicitly stated no blinding of participants or staff, rated as high risk of bias. The rest did not mention blinding, so their risk was unclear. All studies did not mention allocation concealment or assessor blinding, so these were rated as unclear. All included RCTs had complete outcome data, rated as low risk of bias for reporting. For other biases, none were obvious, but lack of description meant the risk remained unclear. The risk of bias assessment diagram can be found in Figure 2, based on the guidelines in the Cochrane Handbook.

## 4 Outcomes

### 4.1 Left ventricular ejection fraction

**FIGURE 3:**
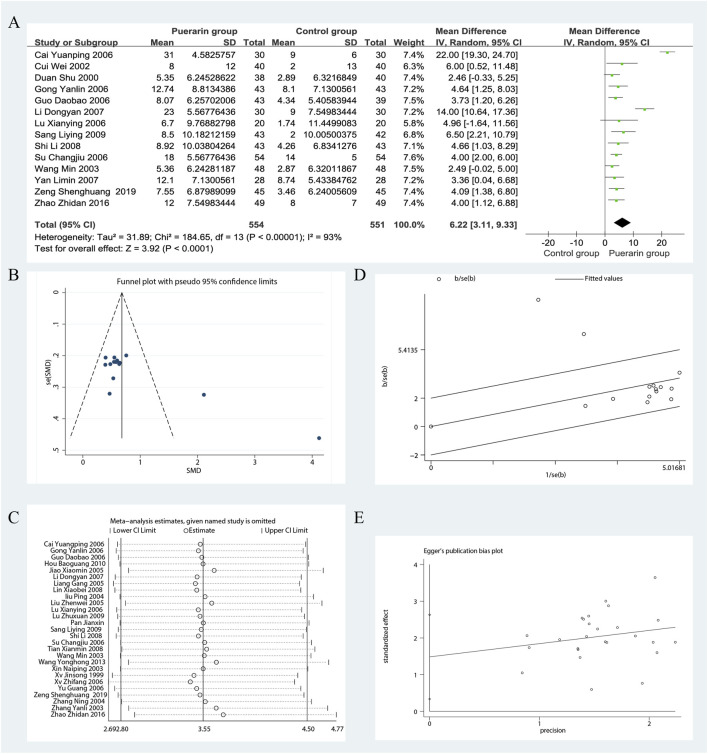
Meta-analysis of LVEF. **(A)** Forest plot; **(B)** Funnel plot; **(C)** Sensitivity analyses; **(D)** Galbraith plot; **(E)** Egger’s test.

Among the 29 studies included, 14 reported on LVEF (Duan et al., 2000; Cui and Xu, 2002; Wang, 2003; Gong and Wang, 2006; Su, 2006; Cai and Liu, 2006; Lu, 2006; Li, 2007; Yan et al., 2007; Shi and Lu, 2008; Sang, 2009; Zhao, 2016; Zeng et al., 2019). These studies included a total of 1,105 patients, with 554 in the treatment group and 551 in the control group. Heterogeneity testing revealed significant heterogeneity among the trials (*P* < 0.00001, *I* ^2^ = 93%), necessitating the use of a random effects model for analysis. The results indicated that the puerarin group showed superior improvement in LVEF compared to the control group [MD = 6.22, 95% CI (3.11, 9.33), *Z* = 3.92, *P* < 0.01], with statistical significance, as illustrated in Figure 3.

To evaluate potential publication bias, we analyzed 14 RCTs using funnel plots, which indicated the presence of asymmetry. In the Galbraith plot, most data points are clustered around the fitted line, indicating a good model fit. However, two data points (Cai and Liu, 2006; Li, 2007) deviate from the fitted line, which may suggest a potential source of heterogeneity. The results of the Egger’s test (*P* = 0.001) indicate significant publication bias. We performed sensitivity analyses using Stata 12.0 software. After excluding certain studies, the point estimates of effect size showed slight deviations from the confidence intervals, suggesting that these studies could influence the results under specific conditions, as illustrated in Figure 3.

**FIGURE 4:**
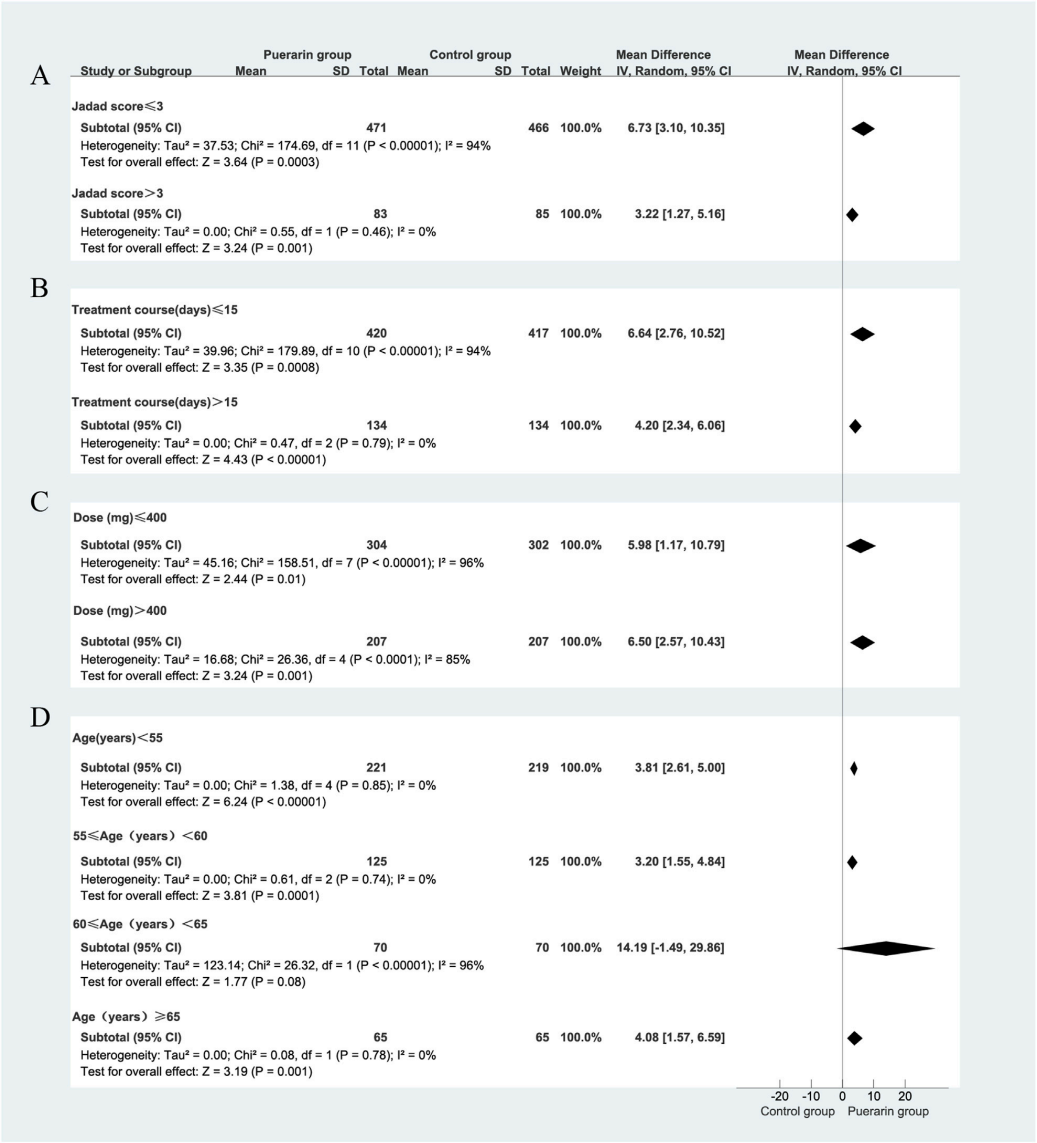
Subgroup analysis of LVEF. **(A)** Jadad score; **(B)** Treatment duration; **(C)** Puerarin injection dosage; **(D)** Average age.

Given the high heterogeneity in the meta-analysis, we performed subgroup analyses to identify its potential sources, focusing on puerarin injection dosage, treatment duration, average age and Jadad score. However, some results still showed significant heterogeneity, suggesting these factors may not be the primary sources. Further research is needed to explore and validate the specific reasons for this heterogeneity, as shown in Figure 6. Notably, the results from the subgroup analysis based on age indicated that for participants with an average age of less than 55 years, the studies exhibited significant homogeneity (*P* = 0.85, *I* ^2^ = 0%). The puerarin group showed a statistically significant improvement in LVEF compared to the control group [MD = 3.81, 95% CI (2.61, 5.00), *Z* = 6.24, *P* < 0.01]. Similarly, for participants aged between 55 and 60 years, there was significant homogeneity (*P* = 0.74, *I* ^2^ = 0%), with the puerarin group again demonstrating significant improvement in LVEF compared to the control group [MD = 3.20, 95% CI (1.55, 4.84), *Z* = 3.81, *P* < 0.01]. In the cohort aged 65 years and older, significant homogeneity was also observed (*P* = 0.78, *I* ^2^ = 0%), with the puerarin group outperforming the control group in terms of LVEF improvement [MD = 4.08, 95% CI (1.57, 6.59), *Z* = 3.19, *P* < 0.01]. However, for participants aged between 60 and 65 years, significant heterogeneity was noted (*P* < 0.00001, *I* ^2^ = 96%), and there was no statistically significant difference between the two groups [MD = 14.19, 95% CI (−1.49, 29.86), *Z* = 1.77, *P* = 0.08]. This suggests that age may influence the relationship between study variables and observed outcomes, particularly in the 60–65 age range, or that other confounding factors may affect the relationship in older populations, as illustrated in Figure 4.

### 4.2 Total effective rate

**FIGURE 5:**
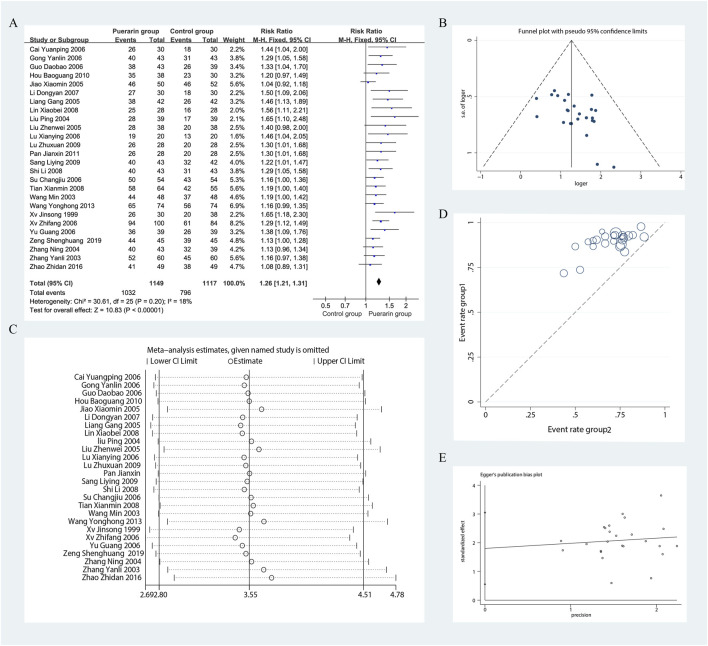
Meta-analysis of total effective rate. **(A)** Forest plot; **(B)** Funnel plot; **(C)** Sensitivity analyses; **(D)** L’Abbe plot; **(E)** Egger’s test.

Among the 29 studies included, 26 assessed the total effective rate as an outcome measure (Xu, 1999; Zhang, 2003; Wang, 2003; Liu et al., 2004; Zhang and Lin, 2004; Liu et al., 2005; Liang et al., 2005; Jiao, 2005; Yu and Liu, 2006; Gong and Wang, 2006; Xu and Zhao, 2006; Su, 2006; Cai and Liu, 2006; Guo et al., 2006; Lu, 2006; Li, 2007; Lin et al., 2008; Tian, 2008; Shi and Lu, 2008; Sang, 2009; Lu, 2009; Hou, 2010; Pan, 2011; Wang and Xie, 2013; Zhao, 2016; Zeng et al., 2019). These studies included a total of 2,266 patients, with 1,149 in the treatment group and 1,117 in the control group. Heterogeneity testing indicated homogeneity among the studies (*P* = 0.20, *I* ^2^ = 18%), allowing for the use of a fixed-effect model in the analysis. The results demonstrated that the puerarin group showed a superior improvement in the total effective rate compared to the control group, with statistically significant differences [RR = 1.26, 95% CI (1.21, 1.31), *Z* = 10.83, *P* < 0.01], as illustrated in Figure 5.

Using funnel plots, we evaluated the potential publication bias in the 26 RCTs. The results indicated slight asymmetry. The L'Abbe plot demonstrated that the puerarin group had a superior improvement in the total effective rate compared to the control group. This distribution suggests a degree of heterogeneity among the different studies or observations. We conducted sensitivity analyses using Stata 12.0 software and found that removing individual studies resulted in slight variations in the combined risk ratio; however, the range of the combined effect size remained consistent, indicating that the results were not sensitive to the exclusion of any single study and exhibited a degree of stability. Nevertheless, results from Egger’s test (*P* = 0.007) indicated the presence of potential publication bias, as illustrated in Figure 5.

**FIGURE 6:**
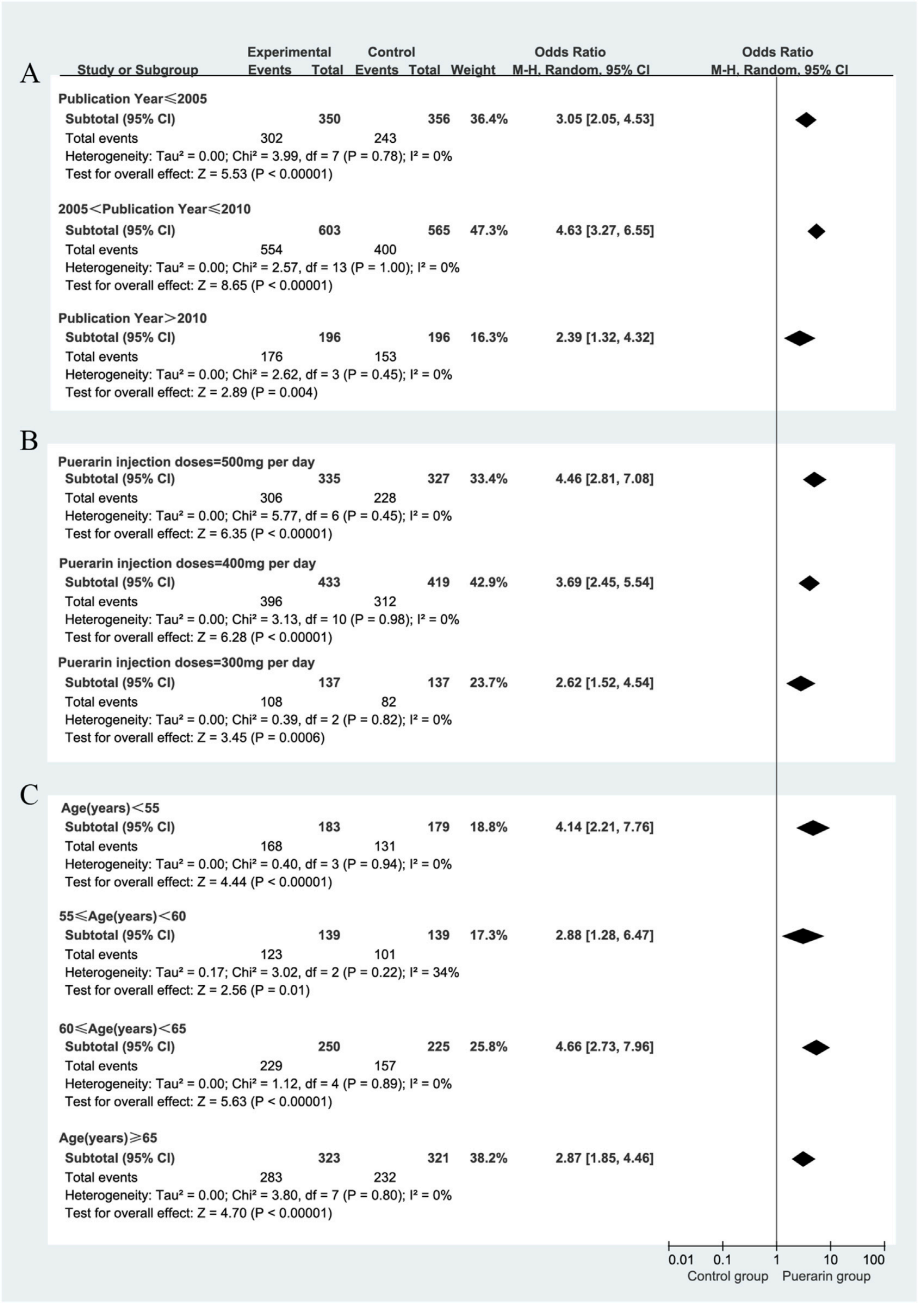
Subgroup analysis of total effective rate. **(A)** Publication year; **(B)** Puerarin injection dosage; **(C)** Average age.

We focused on the publication year of the studies, the dosage of puerarin injection, and average age. However, these factors were unlikely to be major sources of bias. Notably, during the subgroup analysis of puerarin dosage, the 400 mg/day group showed [*P* = 0.98, *I* ^2^ = 0%, RR = 3.69, 95% CI (2.45, 5.54), *Z* = 6.28, *P* < 0.01]. The 400 mg/day dosage demonstrated higher homogeneity across studies compared with the 300 mg/day and 500 mg/day dosages, as illustrated in Figure 6.

### 4.3 Cardiac output

**FIGURE 7:**
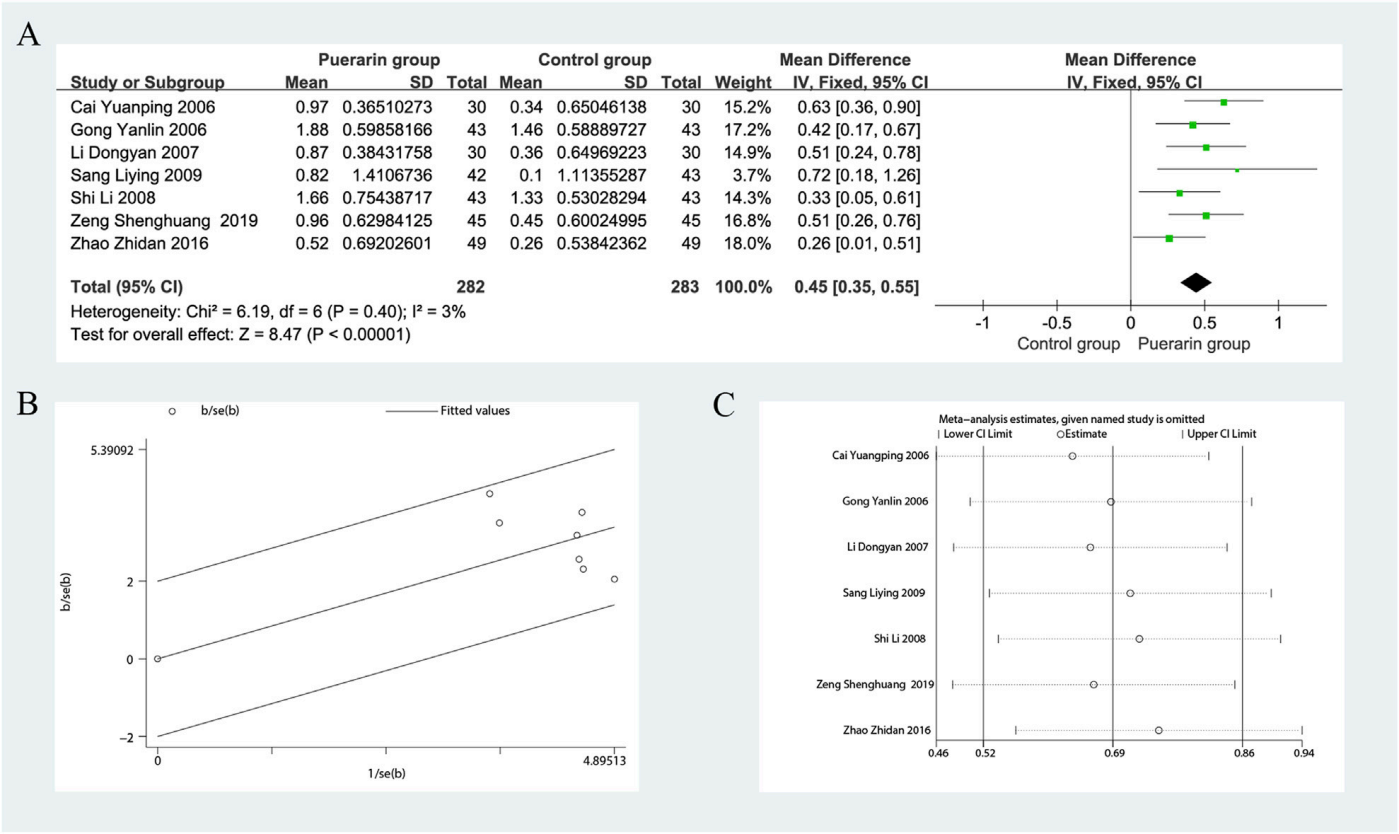
Meta-analysis of CO. **(A)** Forest plot; **(B)** Galbraith plot; **(C)** Sensitivity analyses.

Seven studies reported on CO (Gong and Wang, 2006; Cai and Liu, 2006; Li, 2007; Shi and Lu, 2008; Sang, 2009; Zhao, 2016; Zeng et al., 2019), including a total of 565 patients, with 282 in the treatment group and 283 in the control group. The heterogeneity test indicated homogeneity among the studies (*P* = 0.40, *I* ^2^ = 3%), allowing for the use of a fixed-effects model in the analysis. The results demonstrated that the puerarin group significantly improved CO compared to the control group [MD = 0.45, 95% CI (0.35, 0.55), *Z* = 8.47, *P* < 0.01], indicating statistical significance. The Gabriel plot indicated that as precision increased, the effect size also increased, although a few data points were not aligned with the fitted line, suggesting some heterogeneity. Sensitivity analysis using Stata 12.0 revealed that excluding any single study did not substantially affect the overall effect estimate, indicating that this meta-analysis has good robustness, as illustrated in Figure 7.

### 4.4 Left ventricular end-diastolic diameter

**FIGURE 8:**
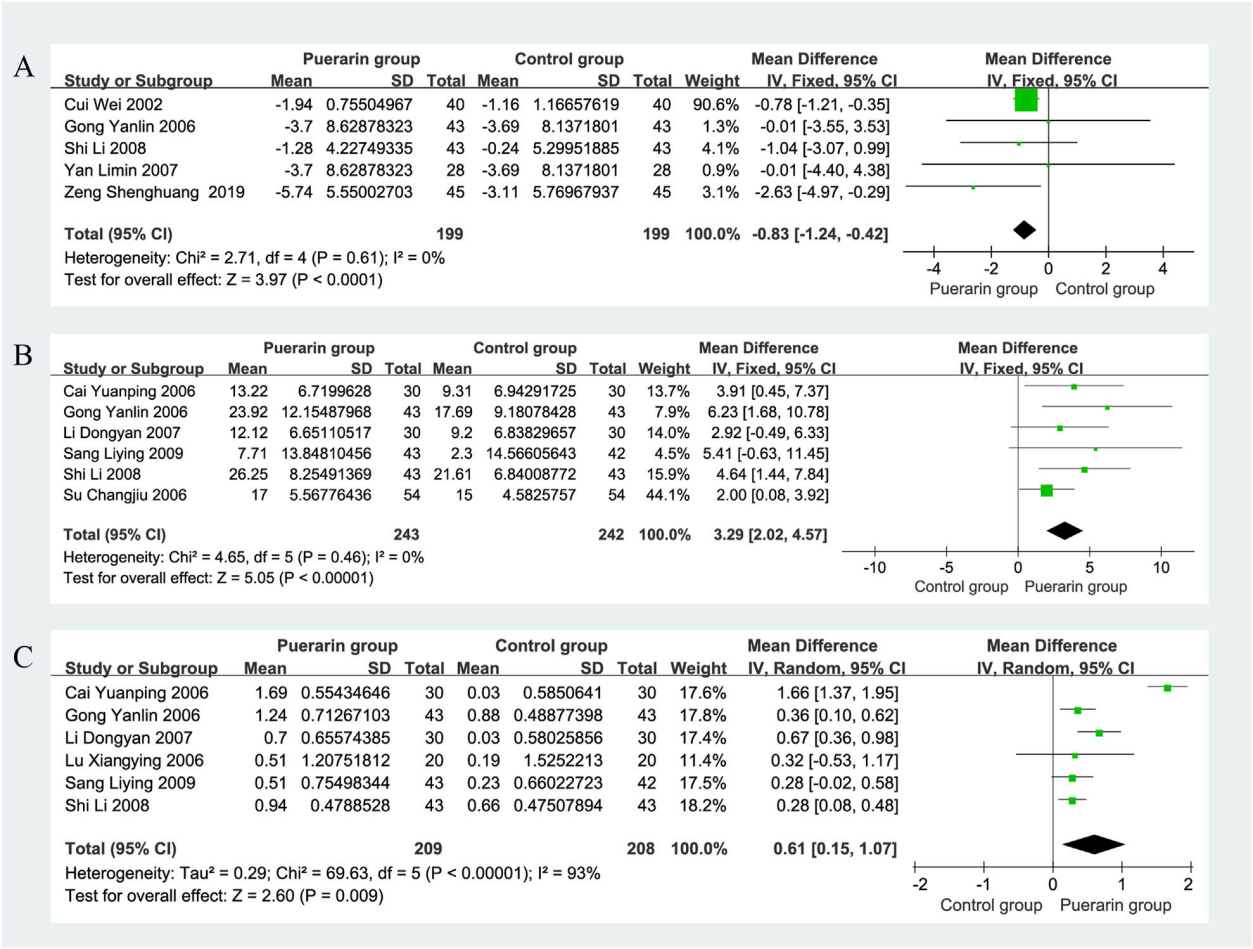
Forest plot. **(A)** LVEDD; **(B)** SV; **(C)** CI.

Five studies reported on LVEDD (Cui and Xu, 2002; Gong and Wang, 2006; Yan et al., 2007; Shi and Lu, 2008; Zeng et al., 2019), involving a total of 398 patients, with 199 in the puerarin group and 199 in the control group. The heterogeneity test indicated homogeneity among the studies (*P* = 0.61, *I* ^2^ = 0%), allowing for the use of a fixed-effects model in the analysis. The results showed that the difference in LVEDD between the puerarin group and the control group was [MD = −0.83, 95% CI (−1.24, 0.42), *Z* = 3.97, *P* < 0.01]. This suggests that puerarin may effectively enhance LVEDD in patients with CHF, as illustrated in Figure 8A.

### 4.5 Left ventricular stroke volume

For SV analysis, six studies (Gong and Wang, 2006; Su, 2006; Cai and Liu, 2006; Li, 2007; Shi and Lu, 2008; Sang, 2009) involving 485 individuals, with 243 in the puerarin group and 242 in the control group. The heterogeneity test indicated homogeneity among the studies (*P* = 0.46, *I* ^2^ = 0%), allowing for the use of a fixed-effects model in the analysis. The results showed that the puerarin group significantly improved SV compared to the control group [MD = 3.29, 95% CI (2.02, 4.57), *Z* = 5.05, *P* < 0.01], with a statistically significant difference. These findings indicate that puerarin appears to be effective in increasing SV in patients with chronic heart failure (CHF), as depicted in Figure 8B.

### 4.6 Cardiac index

Data on CI were reported in six studies (Gong and Wang, 2006; Cai and Liu, 2006; Lu, 2006; Li, 2007; Shi and Lu, 2008; Sang, 2009), which included a total of 417 patients, 209 in the puerarin group and 208 in the control group. The heterogeneity test indicated significant heterogeneity among the studies (*P* < 0.01, *I* ^2^ = 93%), necessitating the use of a random-effects model for analysis. The results showed that the puerarin group significantly improved CI compared to the control group [MD = 0.61, 95% CI (0.15, 1.07), *Z* = 2.60, *P* < 0.01], indicating a statistically significant difference. This suggests that puerarin may effectively enhance CI in patients with CHF, as illustrated in Figure 8C.

### 4.7 Hemorheological indicators

#### 4.7.1 Low shear viscosity

**FIGURE 9:**
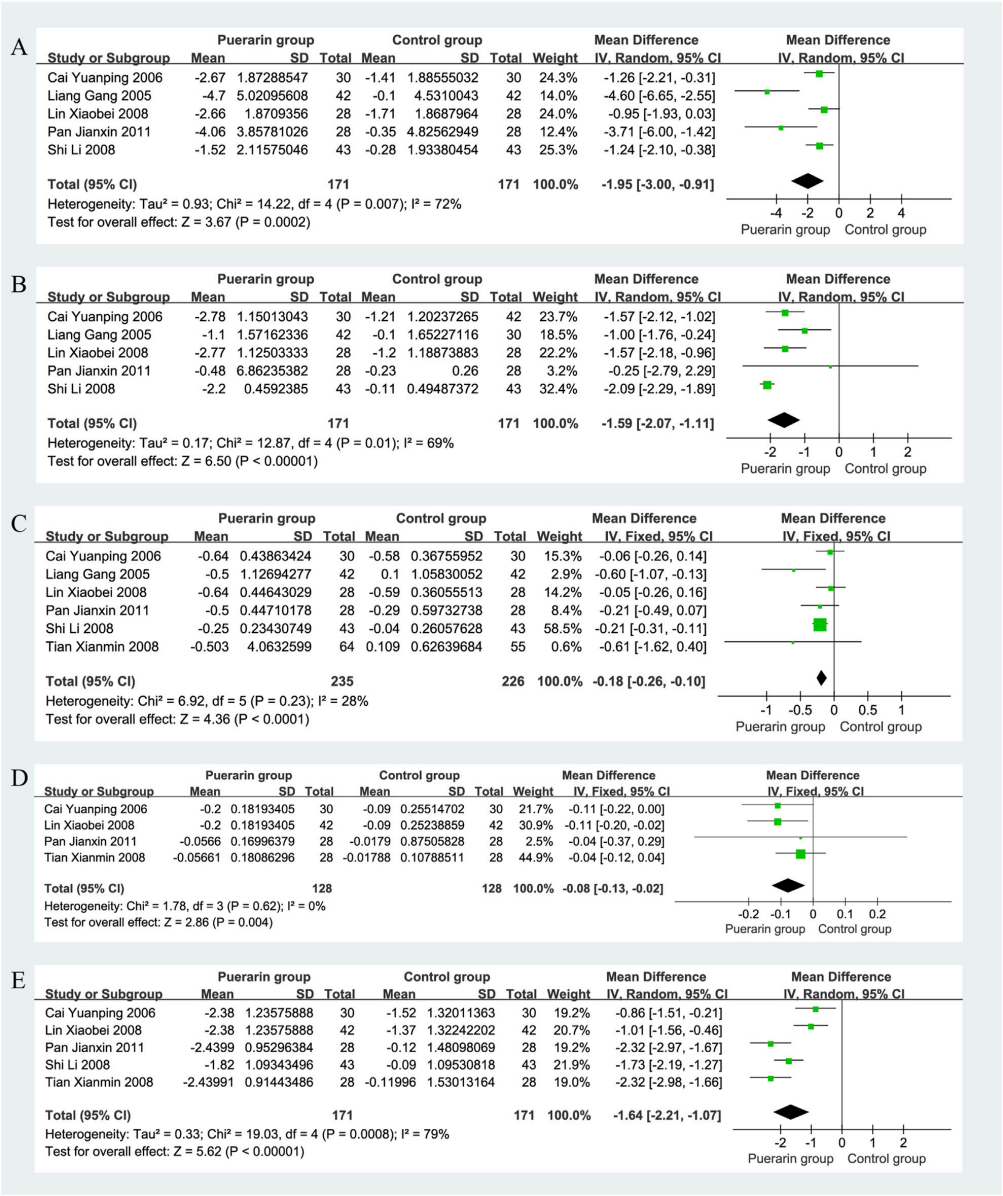
Forest plot. **(A)** Low shear viscosity; **(B)** High shear viscosity; **(C)** Plasma viscosity; **(D)** Platelet aggregation rate; **(E)** Fibrinogen.

Five studies (Liang et al., 2005; Cai and Liu, 2006; Lin et al., 2008; Shi and Lu, 2008; Pan, 2011), which included a total of 342 patients (171 in the puerarin group and 171 in the control group), reported on low shear viscosity. The heterogeneity test showed significant heterogeneity among the studies (*P* = 0.007, *I* ^2^ = 72%), thus a random-effects model was employed for analysis. The results indicated that the puerarin group significantly improved low shear viscosity compared to the control group [MD = −1.95, 95% CI (−3.00, −0.91), *Z* = 3.67, *P* < 0.01], demonstrating a statistically significant difference, as illustrated in Figure 9A.

#### 4.7.2 High shear viscosity

High shear viscosity was reported in five studies (Liang et al., 2005; Cai and Liu, 2006; Lin et al., 2008; Shi and Lu, 2008; Pan, 2011), involving 342 patients with equal group sizes. The heterogeneity test indicated homogeneity among the studies (*P* = 0.01, *I* ^2^ = 69%), allowing for a random-effects model analysis. The results revealed that the puerarin group significantly improved high shear viscosity compared to the control group [MD = −1.59, 95% CI (−2.07, −1.11), *Z* = 6.50, *P* < 0.01], indicating a statistically significant difference, as illustrated in Figure 9B.

#### 4.7.3 Plasma viscosity

Six studies reported on plasma viscosity (Liang et al., 2005; Cai and Liu, 2006; Lin et al., 2008; Tian, 2008; Shi and Lu, 2008; Pan, 2011), with a total of 461 patients, 235 in the puerarin group and 226 in the control group. The heterogeneity test indicated homogeneity (*P* = 0.23, *I* ^2^ = 28%), so a fixed-effects model was used. The results showed that the puerarin group significantly improved plasma viscosity compared to the control group [MD = −0.18, 95% CI (−0.26, −0.10), *Z* = 4.36, *P* < 0.01], indicating a statistically significant difference, as illustrated in Figure 9C.

#### 4.7.4 Platelet aggregation rate

Four studies (Cai and Liu, 2006; Lin et al., 2008; Tian, 2008; Pan, 2011), involving 256 patients (128 in each group), reported on platelet aggregation rate. The heterogeneity test showed homogeneity among the studies (*P* = 0.62, *I* ^2^ = 0%), allowing for a fixed-effects model analysis. The results indicated that the puerarin group significantly improved platelet aggregation rate compared to the control group [MD = −0.08, 95% CI (−0.13, −0.02), *Z* = 2.86, *P* < 0.01], demonstrating a statistically significant difference, as illustrated in Figure 9D.

#### 4.7.5 Fibrinogen

Five studies reported on fibrinogen (Cai and Liu, 2006; Lin et al., 2008; Tian, 2008; Shi and Lu, 2008), also involving a total of 342 patients, with equal group sizes. The heterogeneity test indicated significant heterogeneity among the studies (*P* = 0.0008, *I* ^2^ = 79%), thus a random-effects model was employed for analysis. The results showed that the puerarin group significantly improved fibrinogen levels compared to the control group [MD = −1.64, 95% CI (−2.21, −1.07), *Z* = 5.62, *P* < 0.01], indicating a statistically significant difference, as illustrated in Figure 9E.

### 4.8 Oxidative stress status

#### 4.8.1 Superoxide dismutase

**FIGURE 10:**
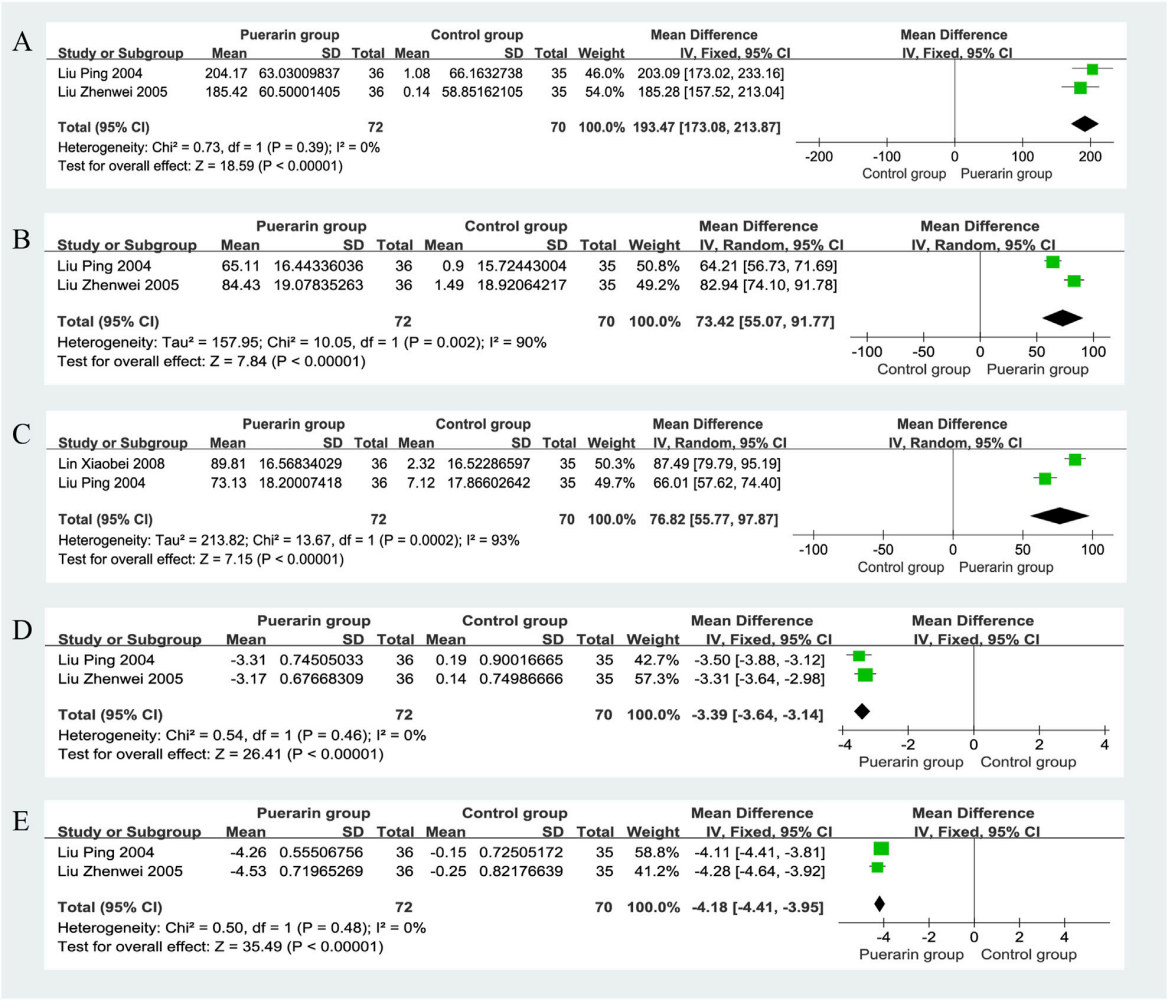
Forest plot. **(A)** SOD; **(B)** GSH-Px; **(C)** CAT; **(D)** LPO; **(E)** MDA.

Two studies reported on Superoxide Dismutase (SOD) (Liu et al., 2004; Liu et al., 2005), involving a total of 142 patients, with 72 in the puerarin group and 70 in the control group. The heterogeneity test indicated homogeneity among the studies (*P* = 0.39, *I* ^2^ = 0%), allowing for a fixed-effects model analysis. The results showed that the puerarin group significantly improved SOD levels compared to the control group [MD = 193.47, 95% CI (173.08, 213.87), *Z* = 18.59, *P* < 0.01], indicating a statistically significant difference, as illustrated in Figure 10A.

#### 4.8.2 Glutathione peroxidase

Two studies (Liu et al., 2004; Liu et al., 2005), which included a total of 142 patients, provided data on Glutathione Peroxidase (GSH-Px). The heterogeneity test revealed significant heterogeneity among the studies (*P* = 0.002, *I* ^2^ = 90%), thus a random-effects model was used for analysis. The results indicated that the puerarin group significantly improved GSH-Px levels compared to the control group [MD = 73.42, 95% CI (55.07, 91.77), *Z* = 7.84, *P* < 0.01], demonstrating a statistically significant difference, as illustrated in Figure 10B.

#### 4.8.3 Catalase

Two studies reported on Catalase (CAT) (Liu et al., 2004; Liu et al., 2005), involving a total of 142 patients. The heterogeneity test indicated significant heterogeneity among the studies (*P* = 0.0002, *I* ^2^ = 93%), thus a random-effects model was employed for analysis. The results showed that the puerarin group significantly improved CAT levels compared to the control group [MD = 76.82, 95% CI (55.77, 97.87), *Z* = 7.15, *P* < 0.01], indicating a statistically significant difference, as illustrated in Figure 10C.

#### 4.8.4 Plasma lipid peroxides

Data on Plasma Lipid Peroxides (LPO) were reported in two studies (Liu et al., 2004; Liu et al., 2005), involving a total of 142 patients, with 72 in the puerarin group and 70 in the control group. The heterogeneity test indicated homogeneity among the studies (*P* = 0.46, *I* ^2^ = 0%), allowing for a fixed-effects model analysis. The results showed that the puerarin group significantly improved LPO levels compared to the control group [MD = −3.39, 95% CI (−3.64, −3.14), *Z* = 26.41, *P* < 0.01], indicating a statistically significant difference, as illustrated in Figure 10D.

#### 4.8.5 Malondialdehyde

Two studies reported on Malondialdehyde (MDA) (Liu et al., 2004; Liu et al., 2005), also involving a total of 142 patients. The heterogeneity test revealed homogeneity among the studies (*P* = 0.48, *I* ^2^ = 0%), thus a fixed-effects model was used for analysis. The results indicated that the puerarin group significantly improved MDA levels compared to the control group [MD = −4.18, 95% CI (−4.41, −3.95), *Z* = 35.49, *P* < 0.01], demonstrating a statistically significant difference, as illustrated in Figure 10E.

### 4.9 Adverse reaction analysis of puerarin injection combined with conventional western medicine

**FIGURE 11:**
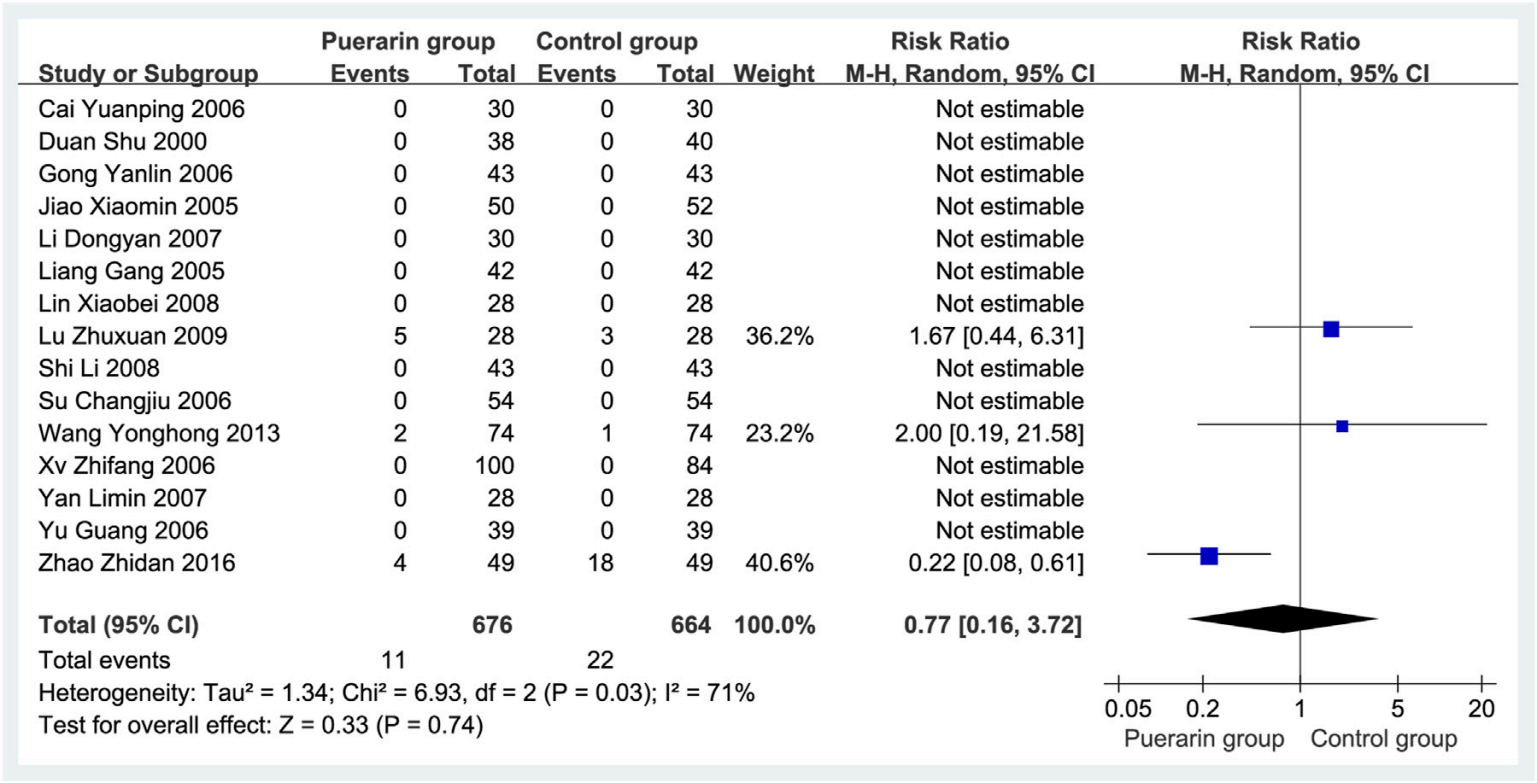
Forest plot of adverse reactions.

A total of 15 studies (Duan et al., 2000; Liang et al., 2005; Jiao, 2005; Yu and Liu, 2006; Gong and Wang, 2006; Xu and Zhao, 2006; Su, 2006; Cai and Liu, 2006; Li, 2007; Yan et al., 2007; Lin et al., 2008; Shi and Lu, 2008; Lu, 2009; Wang and Xie, 2013; Zhao, 2016) reported adverse reactions, involving 1,340 patients (676 in the puerarin group vs. 664 in the control group). The adverse reaction rates were 1.62% (11/676) in the puerarin group and 3.31% (22/664) in the control group, as shown in Table 2. Meta-analysis indicated significant heterogeneity (*P* = 0.03, *I* ^2^ = 71%), necessitating the use of a random-effects model. No statistically significant difference was observed between groups [RR = 0.77, 95% CI (0.16, 3.72), *Z* = 0.33, *P* = 0.74], as illustrated in Figure 11.

**TABLE 2:** The incidence rate of adverse reaction.

Adverse effect	Studies	Total number of adverse effects	
		Puerarin group	Control group
Headache	Lu (2009)	5	3
Ventricular premature contractions	Zhao (2016)	0	5
Conduction block	Zhao (2016)	0	1
Gastrointestinal adverse effects	Wang and Xie (2013), Zhao (2016)	2	9
Respiratory adverse reactions	Wang and Xie (2013), Zhao (2016)	4	4
No adverse effect	Duan et al. (2000), Liang et al., 2005; Jiao (2005), Yu and Liu (2006), Gong and Wang (2006), Xu and Zhao, 2006; Su (2006), Cai and Liu, 2006; Li (2007), Yan et al. (2007), Lin et al. (2008), Shi and Lu (2008)	-	-
Total events	-	11/676	22/664
Incident rate	-	1.62%	3.31%

### 4.10 The quality of the evidence

Based on the assessment of risk of bias, inconsistency, indirectness, imprecision, and other considerations, we utilized GRADE profiler software to evaluate the evidence from the meta-analysis. The results indicated that the quality of the evidence ranged from very low to moderate. The certainty of the evidence for total effective rate, CO, SV, whole blood viscosity, plasma viscosity, and platelet aggregation rate was rated as moderate. In contrast, LVEF, LVEDD, CI, SOD, LPO, MDA, whole blood viscosity, and adverse effects were assessed as low. GSH-Px, CAT, and fibrinogen were rated as very low, primarily due to significant risks of bias, considerable inconsistency, and marked imprecision. Detailed information is provided in Table 3.

**TABLE 3:** The summary findings by the GRADE methods.

Quality assessment							No of patients		Effect		Quality	Importance
No of studies	Design	Risk of bias	Inconsistency	Indirectness	Imprecision	Other considerations	Experimental group versus Control group	Control	Relative (95% CI)	Absolute		
LVEF (Better indicated by lower values)												
14	Randomised trials	Serious^a^	No serious inconsistency^a^	No serious indirectness	Serious^b^	None	554	551	-	MD 6.22 higher (3.11–9.33 higher)	ÅÅOO LOW	CRITICAL
Total effective rate												
26	Randomised trials	Serious^a^	No serious inconsistency	No serious indirectness	No serious imprecision^a^	None	1,032/1,149 (89.8%)	796/1,117 (71.3%)	RR 1.26 (1.21–1.31)	185 more per 1,000 (from 150 more to 221 more)	ÅÅÅO MODERATE	CRITICAL
								72.1%		187 more per 1,000 (from 151 more to 224 more)		
CO (Better indicated by lower values)												
7	Randomised trials	Serious^a^	No serious inconsistency	No serious indirectness	No serious imprecision^a^	None	282	283	-	MD 0.45 higher (0.35–0.55 higher)	ÅÅÅO MODERATE	IMPORTANT
LVEDD (Better indicated by lower values)												
5	Randomised trials	Serious^a^	No serious inconsistency	No serious indirectness	Serious^c^	None	199	199	-	MD 0.83 lower (1.24–0.42 lower)	ÅÅOO LOW	IMPORTANT
SV (Better indicated by lower values)												
6	Randomised trials	Serious^a^	No serious inconsistency	No serious indirectness	No serious imprecision	none	243	242	-	MD 3.29 higher (2.02–4.57 higher)	ÅÅÅO MODERATE	IMPORTANT
CI (Better indicated by lower values)												
6	Randomised trials	Serious^a^	Serious^b^	No serious indirectness	No serious imprecision	None	209	208	-	MD 0.61 higher (0.15–1.07 higher)	ÅÅOO LOW	IMPORTANT
SOD (Better indicated by lower values)												
2	Randomised trials	Serious^a^	No serious inconsistency	No serious indirectness	Serious^d^	None	72	70	-	MD 193.47 higher (173.08–213.87 higher)	ÅÅOO LOW	IMPORTANT
GSH-Px (Better indicated by lower values)												
2	Randomised trials	Serious^a^	Serious^b^	No serious indirectness	Very serious^d^	None	72	70	-	MD 73.42 higher (55.07–91.77 higher)	ÅOOO VERY LOW	IMPORTANT
CAT (Better indicated by lower values)												
2	Randomised trials	Serious^a^	Serious^b^	No serious indirectness	Very serious^d^	None	72	70	-	MD 76.82 higher (55.77–97.87 higher)	ÅOOO VERY LOW	IMPORTANT
LPO (Better indicated by lower values)												
2	Randomised trials	Serious^a^	No serious inconsistency	No serious indirectness	Serious^d^	None	72	70	-	MD 3.39 lower (3.64–3.14 lower)	ÅÅOO LOW	IMPORTANT
MDA (Better indicated by lower values)												
2	Randomised trials	Serious^a^	No serious inconsistency	No serious indirectness	Serious^d^	None	72	70	-	MD 4.18 lower (4.41–3.95 lower)	ÅÅOO LOW	IMPORTANT
Whole blood viscosity (low cut) (Better indicated by lower values)												
5	Randomised trials	Serious^a^	Serious^b^	No serious indirectness	No serious imprecision^c^	None	171	171	-	MD 1.95 lower (3–0.91 lower)	ÅÅOO LOW	IMPORTANT
Whole blood viscosity (high cut) (Better indicated by lower values)												
5	Randomised trials	Serious^a^	No serious inconsistency	No serious indirectness	No serious imprecision	None	171	171	-	MD 1.59 lower (2.07–1.11 lower)	ÅÅÅO MODERATE	IMPORTANT
Plasma viscosity (Better indicated by lower values)												
6	Randomised trials	Serious^a^	No serious inconsistency	No serious indirectness	No serious imprecision	None	235	226	-	MD 0.18 lower (0.26–0.1 lower)	ÅÅÅO MODERATE	IMPORTANT
Platelet aggregation rate (Better indicated by lower values)												
4	Randomised trials	Serious^a^	No serious inconsistency	No serious indirectness	No serious imprecision	None	128	128	-	MD 0.08 lower (0.13–0.02 lower)	ÅÅÅO MODERATE	IMPORTANT
Fibrinogen (Better indicated by lower values)												
5	Randomised trials	Serious^a^	Serious^b^	No serious indirectness	Serious^c^	None	171	171	-	MD 1.64 lower (2.21–1.07 lower)	ÅOOO VERY LOW	IMPORTANT
Adverse reaction												
15	Randomised trials	serious^a^	No serious inconsistency	No serious indirectness	Serious^c^	None	11/676 (1.6%)	22/664 (3.3%)	RR 0.77 (0.16–3.72)	8 fewer per 1,000 (from 28 fewer to 90 more)	ÅÅOO LOW	CRITICAL
								0%		-		

^a^ Absence of blinding.

^b^ High heterogeneity.

^c^ The confidence interval is relatively wide.

^d^ The sample size of the studies included is too small.

## 5 Discussion

### 5.1 Main results of this research

Our systematic review and meta-analysis of 29 RCTs indicated that puerarin injection combined with standard care demonstrated potential advantages over conventional monotherapy in improving multiple outcomes, including LVEF, total effective rate, CO, LVEDD, SV, CI, and hematological/oxidative parameters, without a significant difference in adverse reaction rates between the two groups.

Subgroup analyses on LVEF were performed based on daily dosage, duration of treatment, age and Jadad score. Although the adjunctive interventions improved LVEF, heterogeneity was observed within each subgroup. This suggests that the factors currently considered may not be the primary causes of this heterogeneity. Therefore, future studies should explore and validate the true sources of this variability through additional trials and in-depth research. For participants aged between 60 and 65 years, there was no statistically significant difference between the two groups [MD = 14.19, 95% CI (−1.49, 29.86), *Z* = 1.77, *P* = 0.08]. This suggests that age may influence the relationship between study variables and observed outcomes, particularly in the 60–65 age range. This phenomenon may reflect age-related alterations in pharmacokinetic profiles, aging-related remodeling of target organ receptor systems, and disruption of the metabolic-inflammatory microenvironment, among other factors. We conducted a subgroup analysis on the total effective rate, revealing that puerarin injection doses of 300 mg/day, 400 mg/day, or 500 mg/day all effectively improved total efficacy; however, the 400 mg/day dose exhibited higher homogeneity.

### 5.2 Exploration of the multiple mechanisms of pueraria injection in the treatment of chronic heart failure

In the treatment of CHF, puerarin injection has drawn substantial attention due to its pleiotropic biological activities; however, its precise underlying mechanisms warrant further investigation. By synthesizing the available literature, we have conducted a comprehensive assessment of Gegen’s therapeutic effects. The efficacy of puerarin injection in improving CHF likely stems from its multidimensional cooperative mechanisms involving myocardial protection, functional regulation, and pathological intervention. Molecular insights from preclinical studies demonstrate correlations with our meta-analytical findings, including improvements in cardiac function, ventricular remodeling, hemorheology, and oxidative stress status. Through in-depth exploration of these mechanisms, we can better appreciate the potential advantages and clinical application prospects of puerarin injection in CHF management.

#### 5.2.1 Vasodilatory activity

The vasodilatory effect of puerarin, particularly its endothelium-dependent mechanism, may help reduce peripheral vascular resistance, thereby lowering cardiac afterload. A reduction in afterload means the resistance the heart must overcome during ejection is diminished, which could improve cardiac output and alleviate the workload on the heart. Puerarin exerts an endothelium-dependent vasodilatory effect in the thoracic aorta of rats by activating the BKCa channel (Xh et al., 2007). This activation leads to membrane hyperpolarization, which inhibits voltage-dependent calcium channels, thereby reducing intracellular calcium ion concentration and inducing vasodilation. Studies have shown that puerarin induces vasodilation in a concentration-dependent manner in endothelium-intact aortic rings pre-contracted with deoxycorticosterone or KCl, but does not exhibit this effect in de-endothelialized aortic rings. Moreover, the vasodilatory effect of puerarin is abolished in calcium-free solutions, indicating that its action is calcium-dependent. The anti-vasoconstrictive effect of puerarin is endothelium-dependent and is related to the influx of extracellular calcium into endothelial cells (Zhou et al., 2014). This involves the Ca^2+^-NO-cGMP pathway, the generation of prostacyclin, and the opening of various potassium channels.

#### 5.2.2 Cardiovascular protective effects

Research has demonstrated that puerarin can alleviate ultrastructural changes in the myocardium of diabetic rats, including disorganization of myofibrils and mitochondrial damage, by reducing the expression of *TSP-1* and improving left ventricular function (Pan et al., 2009). Data indicate that puerarin protects endothelial cells from hyperglycemia-induced apoptosis by upregulating *HO-1* expression and inhibiting calpain activity. Additionally, puerarin shows cardioprotective effects in sepsis-induced myocardial injury by activating the AMPK signaling pathway to inhibit ferroptosis (Bin et al., 2022). Furthermore, puerarin effectively enhances myocyte viability and improves mitochondrial function (Shi et al., 2023).

#### 5.2.3 Anti-inflammatory activity

Puerarin exhibits anti-inflammatory effects through multiple signaling pathways, which are not limited to endothelial cells but also affect other cell types, demonstrating its broad applicability in various inflammatory states. This may contribute to improving the inflammatory response associated with heart failure. Puerarin induces HO-1 expression via the PKC δ-Nrf2-HO-1 pathway, showcasing anti-inflammatory properties (Kim et al., 2010). It reduces NF-κB p65 mRNA expression, lowering inflammatory cytokines (TNF-α, MIP-2) (Jj et al., 2012), and inhibits IKKβ/NF-κB activation, decreasing TNF-α and IL-6 production in endothelial cells (Huang et al., 2012). Additionally, puerarin suppresses IL-8 in co-cultures of bronchial epithelial cells and neutrophils (Pang et al., 2012) and protects against ischemia/reperfusion injury by downregulating inflammatory factors (Feng et al., 2014). It inhibits apoptosis and inflammation in cardiac myocytes by upregulating PPARα, reducing LDH, SDH, and inflammatory markers (He et al., 2019). Puerarin also suppresses inflammation by downregulating NF-κB and reducing pro-inflammatory mediators (Jeon et al., 2020). Furthermore, it inhibits ferroptosis through the SIRT1/Nrf2 pathway, improving fatty liver disease (Yang et al., 2023), and blocks NLRP3-Caspase-1-GSDMD-mediated pyroptosis in H9C2 and RAW264.7 cells (Sun et al., 2023).

#### 5.2.4 Antioxidant activity

Puerarin inhibits Cu^2+^-induced LDL oxidation *in vitro* (Liu et al., 2007). It enhances the cell’s antioxidant defense by inducing HO-1 expression through the ER-dependent Gβ1/PI3K/Akt-Nrf2 signaling pathway, protecting cells from oxidative stress damage (Yp and Hg, 2008). The nitrite-glucose-glucose oxidase system can induce protein nitration and oxidation in cardiac homogenates via different pathways, while puerarin exerts its antioxidant effect by inhibiting protein nitration (Naihao et al., 2009). Puerarin-7-O-glucuronide significantly alleviates hypertrophy in cultured cardiac myocytes by improving oxidative stress (Hou et al., 2017). It demonstrates antioxidant activity by modulating the Nrf2 pathway and the expression of antioxidant enzymes (Jeon et al., 2020). This mechanism is significant for protecting cardiac myocytes and enhancing their function, suggesting the potential applications of puerarin in addressing oxidative stress-related cardiac diseases.

#### 5.2.5 Improvement of cellular autophagy

Puerarin restores autophagy via the AMPK/mTOR-mediated signaling pathway, partially contributing to its protective effects against cardiac myocyte hypertrophy and apoptosis (Bei et al., 2015). Puerarin and Torin1 can significantly inhibit the progression of sepsis by regulating mitochondrial autophagy-related proteins *p62*, *LC3B*, *Pink1*, and *Parkin*, thereby reversing lipopolysaccharide-induced suppression of mitochondrial autophagy in H9C2 cardiac cells (Chang et al., 2022). Puerarin protects human bronchial epithelial cells (HBECs) by activating the *PI3K/AKT/mTOR* signaling pathway to inhibit mitochondrial autophagy induced by cysteamine (CSE) (Wang et al., 2022). Additionally, puerarin-mediated autophagy activation can induce an increase in *PPARβ/δ* expression, significantly promoting angiogenesis in vascular endothelial cells (Pan et al., 2023). This highlights the importance of puerarin in regulating autophagy, which may provide new insights for the treatment of heart failure.

#### 5.2.6 Inhibition of ferroptosis

Ferroptosis is characterized by iron overload, dependent on iron ions and LPO, leading to disruption of cell membrane integrity and ultimately cell death, closely linked to the onset and progression of heart failure (Xiaoguang et al., 2021). Puerarin reduces cardiac myocyte loss during heart failure by partially inhibiting ferroptosis, suggesting its potential as a novel therapeutic approach for heart failure (Liu et al., 2018). Puerarin exerts anti-inflammatory effects in lipopolysaccharide-induced RAW264.7 macrophages by modulating ferroptosis-related pathways, including arachidonic acid metabolism, tryptophan metabolism, and glutathione metabolism (Jinzi et al., 2022). Furthermore, puerarin mitigates sepsis-induced myocardial injury by activating the AMPK signaling pathway to inhibit the ferroptosis process (Bin et al., 2022). Puerarin demonstrates its potential as a novel mechanism for heart failure treatment by inhibiting ferroptosis and reducing cardiac myocyte loss. Its ability to regulate related metabolic pathways suggests that puerarin may have unique advantages in clinical applications.

### 5.3 Safety of puerarin injection combined with conventional drug therapy in the treatment of chronic heart failure

In the Ames test (Chung et al., 2009), puerarin and its glycosides did not show mutagenic effects at concentrations up to 200 μg/plate. In a bone marrow micronucleus test conducted using ICR mice, neither puerarin nor glucose-α-(1,6)-puerarin interfered with erythropoiesis in the bone marrow. Sprague-Dawley rats receiving daily oral doses of puerarin and its glycosides for 28 days showed no significant changes in histological, biochemical, or hematological parameters. These results indicate that, at doses up to 250 mg/kg/day, puerarin and its glycosides do not exhibit significant toxicity in rodents, whether *in vitro* or *in vivo*. However, embryos treated with 5 or 10 μM puerarin showed a significantly increased apoptosis rate and a marked reduction in total cell number (Chen and Chan, 2009). Interestingly, there was no significant difference in implantation success rates between puerarin-pretreated embryos and the control group, but *in vitro* treatment with 5 or 10 μM puerarin was associated with increased embryo resorption and decreased fetal weight in mice after implantation. *In vitro* exposure to puerarin induces apoptosis and delays early post-implantation development when transferred to host mice. Puerarin crosses the placenta, maintaining high concentrations in fetal plasma, which may lead to impaired embryonic development and viability (Huang and Chan, 2016), suggesting caution in its use during pregnancy.

A study evaluated the safety of intravenous infusion of puerarin injection (Xie et al., 2018), reporting that adverse reactions, primarily allergic reactions and acute intravascular hemolysis, accounted for 88.3% of cases. In our study, the incidence of adverse reactions in the puerarin group was 1.62% (11 out of 676). The reported adverse reactions included headache, gastrointestinal reactions, and respiratory system reactions. No statistically significant difference was observed compared to the control group, and no serious adverse events were reported. However, the quality of the included RCTs was poor, and the quality evaluation of the meta-analysis was rated as LOW. Therefore, we can only preliminarily conclude that puerarin injection is safe as an adjunctive treatment for CHF, indicating that further high-standard clinical research is needed to validate and confirm our findings. Puerarin is classified as an acidic TCM injection, and clinical use should strictly adhere to indications and administration methods (Li et al., 2014). When used for the indications specified in the prescribing information, the adverse reaction severity is milder when 5% glucose injection is used as a solvent. Careful inquiry into patients’ allergy histories and enhanced monitoring during treatment are recommended.

### 5.4 Dosage and treatment duration of puerarin injection as an adjunctive therapy for chronic heart failure

The dosage and treatment duration of puerarin injection for CHF remain subjects of debate. Our study results indicate that the most commonly selected daily doses of puerarin injection are 400 mg (11/29) and 500 mg (9/29). However, treatment at 400 mg per day yields more stable efficacy. The majority of studies had a treatment duration of 14 days or longer (23/29), achieving favorable outcomes. In conjunction with our subgroup analysis of LVEF, treatment durations exceeding 14 days showed more consistent improvements in LVEF. Therefore, we recommend a dosage of 400 mg per day for puerarin injection, with a minimum treatment duration of 14 days.

## 6 Limitations

Despite achieving favorable results, this study has certain limitations. According to the quality assessment standards outlined in the Cochrane Handbook and the Preferred Reporting Items for Systematic Reviews and Meta-Analyses (PRISMA) statement, the clinical studies we included exhibit deficiencies in aspects such as allocation concealment, blinding of participants and researchers, and outcome assessment blinding. Consequently, the quality of the included studies is low, necessitating the conduct of rigorously designed trials with unified standards to further validate the efficacy of puerarin injection as an adjunctive therapy for CHF. Although no language restrictions were imposed during the literature screening process, all studies ultimately included were conducted in China, which may introduce potential selection bias. The treatment duration, dosage of puerarin injection, and outcome measures varied among the included studies, with some data being unavailable, preventing subgroup analyses to address high heterogeneity. Furthermore, the studies included ranged from 2003 to 2019, during which treatment protocols and medications for heart failure may have evolved. The use of GRADEprofiler software to evaluate the evidence from the meta-analysis revealed that the quality of evidence ranged from moderate to very low. Thus, caution should be exercised when interpreting the results of the meta-analysis in clinical practice. CHF is a condition that requires long-term management, and extended follow-up is essential to observe the long-term benefits of puerarin injection; however, none of the included studies addressed follow-up. Therefore, until further rigorously designed long-term follow-up studies involving diverse populations can provide higher-quality data to update this meta-analysis, we should maintain a cautious stance regarding the existing results.

## 7 Conclusion

The evidence provided by this study indicates that compared with conventional therapy, puerarin injection as an adjunctive treatment for CHF may offer potential advantages in improving overall clinical effectiveness, enhancing cardiac function, ameliorating ventricular remodeling, optimizing hemorheological parameters, and reducing oxidative stress, while exhibiting an acceptable safety profile. However, given the overall low quality of current evidence, future research should employ rigorously designed methodologies including double-blind, large-sample, multicenter trials with long-term follow-up across diverse populations. Such enhanced investigations will enable further validation and updating of existing findings, thereby providing more robust evidence-based medical support for clinical decision-making.

## Data Availability

The original contributions presented in the study are included in the article/Supplementary Material, further inquiries can be directed to the corresponding author.
