# Supplementary material for: Efficacy and safety of puerarin injection as an adjunctive therapy for chronic heart failure: a systematic review and meta-analysis
Source: Front Pharmacol. 2025 Apr 28;16:1516059. doi: 10.3389/fphar.2025.1516059 (PMC12086430; doi:10.3389/fphar.2025.1516059)
Supplement: Supplementary file 1 [file Table1.docx]

# Supplementary Table S1

|  | **Search items** | **Number** |
| --- | --- | --- |
| **PubMed** | ("Puerarin"[Mesh] OR Puerarin[tiab] OR Kakonein[tiab] OR Gegensu[tiab] OR "Puerarin injection"[tiab] OR "Kakonein injection"[tiab]) AND ("Heart Failure"[Mesh] OR "Cardiac Failure"[Mesh] OR (heart failure[tiab] OR cardiac failure[tiab] OR CHF[tiab] OR chronic heart failure[tiab])) | 25 |
| **Embase** | ('puerarin'/exp OR 'puerarin' OR 'kakonein' OR 'puerarin injection') AND ('heart failure' OR 'cardiac failure' OR 'CHF' OR 'chronic heart failure') | 78 |
| **Web of science** | (Puerarin) OR (Kakonein) OR (Puerarin injection) (All Fields) and (Heart Failure) OR (Cardiac Failure) OR (CHF) OR (chronic heart failure) (All Fields) | 34 |
| **Cochrane** | ((Puerarin) OR (Kakonein) OR (Puerarin injection)):ti,ab,kw AND ((Heart Failure) OR (Cardiac Failure) OR (CHF) OR (chronic heart failure)):ti,ab,k | 4 |
| **CNKI** | （主题：葛根素+葛根素注射液）AND（主题：慢性心力衰竭+慢性心衰+心力衰竭+心衰) | 200 |
| **Wanfang** | 主题:(葛根素 OR 葛根素注射液) and 主题:(慢性心力衰竭 or 慢性心衰 or 心衰 or 心力衰竭) | 166 |
| **VIP** | ((题名或关键词=葛根素 OR 题名或关键词=葛根素注射液) AND (((题名或关键词=心力衰竭 OR 题名或关键词=慢性心力衰竭) OR 题名或关键词=慢性心衰) OR 题名或关键词=心衰)) | 106 |
| **SinoMed** | ( "葛根素"[全部字段:智能] OR "葛根素注射液"[全部字段:智能]) AND("心力衰竭"[全部字段:智能] OR "心衰"[全部字段:智能] OR "慢性心力衰竭"[全部字段:智能] OR "慢性心衰"[全部字段:智能]) | 148 |
